# Identifying Host Factors That Regulate Viral Infection

**DOI:** 10.1371/journal.ppat.1002772

**Published:** 2012-07-12

**Authors:** Tien-Huei Hsu, Katherine R. Spindler

**Affiliations:** Department of Microbiology and Immunology, University of Michigan, Ann Arbor, Michigan, United States of America; University of Florida, United States of America

## The Host Side of Viral Infection

One goal of virology research is to identify viral and host factors involved in infection, in order to develop antiviral therapies. Drugs targeting viral proteins have certain key disadvantages. They often affect only a specific viral species or subtype. Also, the low-fidelity polymerases of many medically important viruses, including HIV and influenza, make them prone to rapid mutations, leading to development of drug resistance. In addition, viruses encode few proteins, limiting the number of available targets.

Targeting host proteins is a practical alternative. Viruses use host proteins at multiple stages of their life cycles. Identifying host functions subverted by viruses will further our understanding of viral life cycles and may provide a catalog of novel drug targets that are unlikely to mutate following therapy. Furthermore, targeting the host may result in therapies with a broader range than traditional antivirals. Exciting progress has been made in recent years in this field; the development of new genomic and proteomic tools enables identification of interacting host factors at an unprecedented scale and level of detail. Together with the use of bioinformatics, these approaches hold promise for accelerating our understanding of virus–host interactions.

## Genomics Techniques to Identify Host Factors

Host genetic background can significantly influence the outcome of viral infection. Genetic studies identify host factors required for successful viral infection through phenotypic effects such as susceptibility. The ability to manipulate experimental animals has expanded our knowledge of host factors involved in infection. For example, inbred mice that exhibit inherent phenotypic differences in their susceptibility profiles can be bred to generate progeny whose genotypes and phenotypes can be determined. Linkage analysis tools can then be used to identify a candidate region, and potential disease susceptibility genes can be prioritized for positional cloning.

Through genetic mapping, mouse cytomegalovirus (MCMV) susceptibility was determined to be associated with the loss of an activating natural killer cell receptor [1]. A genetic approach was also used to identify the *Flv* gene, subsequently identified as *Oas1b*, a member of the OAS/RNASEL innate immune system, which is responsible for controlling resistance to West Nile virus infection in mice [2]. A quantitative trait locus (QTL) strongly linked to susceptibility to mouse adenovirus type 1 was identified and reduced rapidly from an 18-Mb region to only 0.75 Mb through positional cloning involving backcross mice, polymorphic markers, and single nucleotide polymorphism haplotype identity [3]. Each of these studies began with the identification of mouse strains with differing susceptibilities to infection. However, due to the small number of inbred mouse strains and the limited genetic diversity of currently available strains, researchers are not able to achieve strong mapping resolution initially and must use additional methods to identify candidate genes. Optimally, researchers will be able to map genetic loci at a resolution that allows identification of individual genes, eliminating the steps of candidate gene prioritization.

To develop a genetically diverse panel of inbred mouse strains to increase mapping resolution, a community effort has created the Collaborative Cross (CC) [4]. In a recent study, 44 pre-CC mouse strains were used to identify 21 QTLs associated with regulation of host response to influenza infection [5]. Pre-CC mice are in the process of becoming inbred CC strains; this study clearly demonstrates that CC mice have greater phenotypic diversity than standard inbred mouse strains. Pre-CC mice were also used to create Diversity Outbred (DO) mice [6]. DO mice are maintained through outcrossing to maintain allelic diversity; CC mice are inbred to generate stable clones. Complementary use of CC and DO mice will allow researchers to identify genes important in complex traits such as susceptibility to viral infection. However, these strategies rely on identifying pre-existing variants in host susceptibility genes.

In contrast, novel germline mutations can be created using mutagens, such as *N*-ethyl-*N*-nitrosourea [7]. MCMV-resistant mice were mutagenized and selected for susceptibility to MCMV. Genes associated with resistance were then identified through positional cloning and sequencing. This same approach was recently used to identify a mouse gene, *Eif2ak4* (encoding GCN2), involved in susceptibility to MCMV and human adenovirus [8]. The advantage of this approach in identifying new host factors and pathways is that it is unbiased and does not make assumptions of the genes involved.

Efforts to determine human homologs of susceptibility genes identified in mouse models are underway to translate these findings to human disease. Mouse studies are an important starting point for uncovering virus–host interactions, especially when orthologous human genes are present. However, human populations are outbred, and variations in response to viral infection are expected, resulting in less than clear interpretation of results. In humans, genome-wide linkage analysis studies have been limited to chronic infectious diseases, due to the difficulty in recruiting families with multi-case acute viral infections. A whole genome scan conducted with Gambian families identified a major susceptibility locus to chronic hepatitis B infection that contains a cluster of cytokine receptor genes [9]. Family-based linkage studies have the additional disadvantage of having low power in identifying genes involved in susceptibility to viral diseases that involve the complex interaction of multiple genes. In addition, family members share many genes, making it difficult to identify the relevant genes involved in viral susceptibility.

Genome-wide association studies (GWAS) have been used to identify human susceptibility loci. Whole genomes of a large human population can be scanned to identify genetic variations frequently associated with susceptibility to infection by a particular pathogen or with severity of disease. The HLA-viral peptide interaction was identified through GWAS as a major genetic factor responsible for HIV control [10]. GWAS require a large sample size and can suffer from sample-selection biases of cases and controls. These studies also have limited ability to detect variants with small effect or low frequencies. However, next generation sequencing will facilitate identification of rare mutations associated with host susceptibility.

## Direct Protein-Based Techniques to Identify Host Factors

Many methods can be used to identify physical interactions between viral and host proteins. One of the earliest of these was co-immunoprecipitation of viral and cellular protein complexes with specific antisera to viral and host proteins. The tumor suppressor protein p53 was first identified by co-immunoprecipitation in complexes with adenovirus E1B 55 kDa protein and in complexes with SV40 large T antigen [11]. The tumor suppressor protein Rb co-immunoprecipitates with adenovirus E1A protein [12]. These findings provided critical evidence that oncogenic viruses promote tumorigenesis by inactivating tumor suppressor proteins. However, co-immunoprecipitation is performed in vitro and may not accurately represent the interaction of proteins in vivo. In addition, weak or less stable interactions may be overlooked.

Additional techniques used to detect interactions of viral and host proteins include yeast-two-hybrid (Y2H), tandem affinity purification, virus overlay protein binding assay (VOPBA), glutathione S-transferase protein purification, and co-immunoprecipitation followed by mass spectrometry analysis. Y2H is amenable to high-throughput screening, and genome-scale Y2H studies have identified host–viral protein interactions for a variety of viruses, including Epstein-Barr virus, HIV, influenza virus, vaccinia virus, Moloney murine leukemia virus, and hepatitis C virus [13]. When the Y2H approach is adapted to high-throughput format, a single “bait” can be tested against multiple “preys” for physical interaction. VOPBA is a screen for interacting proteins using electrophoresis of cellular contents, followed by blotting to a membrane and “probing” with virus. VOPBA has been used to identify virus receptors for human adenovirus [14], respiratory syncytial virus [15], lymphocytic choriomeningitis virus, and Lassa fever virus [16]. Results from Y2H and VOPBA can be validated by co-immunoprecipitations of co-transfected proteins, but the techniques are limited to direct protein–protein interactions.

Gene silencing techniques can assist in defining effects of cellular factors on viral infection that are both direct and indirect. These include host factors (i) that interact directly with viral proteins, (ii) that are present in viral–host protein complexes, (iii) that bind to non-protein components of viruses, and (iv) that are involved in signaling pathways, other cellular processes involved in viral infection, and host immunity. Genome-scale RNA interference (RNAi) screening is a high-throughput method used to investigate diverse biological processes, including host factors involved in viral pathogenesis. Large-scale RNAi screens have been used to identify host factors for a number of important human viruses, including HIV, hepatitis C virus, influenza virus, West Nile virus, and dengue virus [13]. However, because it is technically challenging to develop complete RNAi libraries of the human genome, important candidates may be missed [17]. RNAi screens are highly sensitive to experimental variation, and the overlap of positive hits between similar studies can vary [18]. Also, because RNAi screens are resource intensive, often few time points are examined, limiting knowledge of dynamic changes during viral infection.

Molecular imaging techniques are increasingly being used to visualize transient or dynamic interactions. Live cell imaging microscopy techniques have advanced significantly, allowing detection of single molecules in the absence of artifacts caused by fixation methods. Events of influenza entry have been dissected using real-time microscopy, providing new insights into cellular endocytic pathways [19]. Two different host proteins that interact with the Sindbis virus at different stages of infection were identified using a GFP-tagged viral protein, further demonstrating the usefulness of imaging approaches [20]. Major considerations of this technique are the maintenance of constant physiological conditions (e.g., temperature and pH), and the prevention of photobleaching of dyes. These may prove challenging following extended imaging. However, the ability to monitor rapidly changing interactions may provide critical insight into viral processes that are not readily measured using other methods.

## Data Repositories

Data generated from high-throughput techniques have furthered our understanding of the virus–host interface, and efforts are being made to identify and analyze candidate drug targets. To maximize the benefits of these screens, data need to be accessibly stored and modeled into networks. Several online repositories, including VirHostNet [21], VirusMINT [22], and BiologicalNetworks [23], enable modeling of current data to gain broad understanding of protein and gene networks involved in viral infection. Multi-scale data integration approaches allow for simultaneous analysis of different datasets, such as phylogeny, literature searches, virulence, and epidemiological data. However, there has been no standardization of where data should be deposited, and participation is voluntary.

## Concluding Remarks

Various techniques have facilitated identification of host factors involved in viral infection. Verification of these candidates through biochemical, genetic, and immunological methods may progressively become the rate-limiting step. Virologists will increasingly need to collaborate with other scientists to realize the full potential of the collected data. Use of simulations and models will enable better depiction of infection events. Structural biology can also be used to visualize protein interfaces at high resolution. The identification of host proteins through the many approaches described in this review is only a starting point for exploring function and mechanism, with the aim of uncovering cellular pathways affecting viral replication that can be targeted for drug development.
